# Constrained Flooding Based on Time Series Prediction and Lightweight GBN in BLE Mesh

**DOI:** 10.3390/s24144752

**Published:** 2024-07-22

**Authors:** Junxiang Li, Mingxia Li, Li Wang

**Affiliations:** School of Software, Northwestern Polytechnical University, Xi’an 710072, China; lijunxiang@mail.nwpu.edu.cn (J.L.); limingxia@mail.nwpu.edu.cn (M.L.)

**Keywords:** BLE mesh, low-power, friendship, time series prediction, lightweight GBN

## Abstract

Bluetooth Low Energy Mesh (BLE Mesh) enables Bluetooth flexibility and coverage by introducing Low-Power Nodes (LPNs) and enhanced networking protocol. It is also a commonly used communication method in sensor networks. In BLE Mesh, LPNs are periodically woken to exchange messages in a stop-and-wait way, where the tradeoff between energy and efficiency is a hard problem. Related works have reduced the energy consumption of LPNs mainly in the direction of changing the bearer layer, improving time synchronization and broadcast channel utilization. These algorithms improve communication efficiency; however, they cause energy loss, especially for the LPNs. In this paper, we propose a constrained flooding algorithm based on time series prediction and lightweight GBN (Go-Back-N). On the one hand, the wake-up cycle of the LPNs is determined by the time series prediction of the surrounding load. On the other, LPNs exchange messages through lightweight GBN, which improves the window and ACK mechanisms. Simulation results validate the effectiveness of the Time series Prediction and LlightWeight GBN (TP-LW) algorithm in energy consumption and throughput. Compared with the original algorithm of BLE Mesh, when fewer packets are transmitted, the throughput is increased by 214.71%, and the energy consumption is reduced by 65.14%.

## 1. Introduction

### 1.1. Motivation

In the Internet of Things (IoT), Low-Power Nodes (LPNs) are ideal for building networks for smart homes, smart lighting, building automation, and industrial IoT [1,2,3,4] due to their flexibility. LPNs can be easily deployed anywhere in the network without a fixed power supply [5]. This improves the applicability of the network. Therefore, they are also an excellent choice for sensor networks. As a routing algorithm for BLE Mesh networks, the Constrained Flooding algorithm is used in standard protocols. The constrained flooding algorithm [6,7] introduces Heartbeat messages and Time To Live (TTL) into flooding messages. Heartbeat messages indicate the active state of the node and TTL controls the relay distance of the message. The scope of flooding is restricted by these two messages. However, devices in a BLE Mesh network need to constantly scan the broadcast channel to ensure that they do not miss the packages sent by the flood [8]. This operation significantly reduces the lifetime of the devices [9,10,11]. However, heartbeat messages and TTL have only a limited effect on the size of the flood [2]. Therefore, BLE Mesh limits it further by a friendship mechanism. The LPNs keep themselves asleep most of the time by establishing a friendship relationship with friend nodes. This reduces the energy consumption of the LPN and extends its lifetime.

Although in the BLE Mesh standard protocol the friendship mechanism reduces the energy consumption of LPNs to some extent, there are still some problems. On one hand, it may lead to LPNs establishing a friendship with a friend node of higher load. This will result in LPNs needing to request more frequently or scan for a longer time to complete the data transfer. On the other hand, LPNs use a stop-and-wait protocol for communication in standard protocols. The node sends a packet, waits for an acknowledgment, and then sends the next packet after receiving the acknowledgment. It is a relatively inefficient communication method [12], which is unfavorable for the LPNs to quickly complete the data transfer and enter the sleep state.

### 1.2. Related Work

One way to address the energy consumption of LPNs is to change the bearer layer. A proprietary solution is proposed in the literature [13]. Data transmission using extended broadcasts updated with BLE version 5.0 to improve bandwidth. Although this method can reduce the energy consumption of LPNs, there is a mandatory requirement for the BLE version of the devices in the network.

Another way to reduce the energy consumption of LPNs is to improve time synchronization. The main energy consumption of LPNs comes from the sending and receiving data packets. By improving the time synchronization between the LPN and the friend node, LPNs reduce the number of requests and scanning time. This optimization is implemented in two main ways: one is to use a high precision time synchronization algorithm [14], and the other is to use a high-frequency source to improve the clock accuracy [15]. The asynchronous dynamic scanning algorithm [16] defines a new control command to adjust the scanning parameters of LPNs. When the network needs to transmit data, the scanning window is set to the scanning interval for waiting for data. When the data transmission is completed, the LPNs in the network return to a low-duty cycle scanning state. The BTLBT (Burst Transmissions and Listen Before Transmit) algorithm [12] scans the channel before sending a request during the wake-up cycle of an LPN. If the friend node is sending data to other nodes at this time, it enters a short sleep state while waiting for the friend node to complete the current broadcast event. These two algorithms can improve the transmission efficiency of the friendship mechanism during data transmission, but the time synchronization algorithms and high-frequency sources running in LPNs increase energy consumption.

Other studies have proposed that LPNs send packets only on a single channel. This is because packets in the BLE Mesh network require sending on three broadcast channels sequentially. Sending on a single channel reduces the energy consumption incurred for sending packets [17]. However, the friend node still polls and scans on all three channels, increasing the likelihood of losing friend poll messages. This wastes the scanning window and leads to increased energy consumption in LPNs.

There is also a line of research looking at reducing the overall energy consumption of the network. Novel NonUniform Power Formation Algorithm (NUPFA) [18] builds networks in a distributed manner, utilizing nonuniform power allocation. The overall power consumption is reduced by decreasing the packet transmission power of each small network. The Geographic information and packet delivery probability-based Candidate Set Selection Algorithm (GPCSSA) and Markov Chain-based Prioritization Algorithm (MCPA) [19] reduce the overall energy consumption of the network by selecting the node with the minimum forwarding power per hop in routing. The above two algorithms extend the overall network lifetime but are not optimal solutions for the energy consumption of LPNs.

In summary, current research has been carried out to reduce the energy consumption of BLE networks by changing the bearer layer, improving the clock synchronization, and increasing the broadcast channel utilization. However, we can further optimize the energy consumption of the LPNs. We found that there is a state change in whether LPNs establish friendship relationships or not, which has a certain periodic pattern. Therefore, we consider introducing a time series prediction model to predict the load of nodes. This can improve the transmission efficiency of LPNs while further reducing the energy consumption of LPNs. This paper proposes a constrained flooding algorithm based on time series prediction and the lightweight GBN protocol (TP-LW). The TP-LW algorithm combines load prediction with a lightweight GBN protocol. It reduces the energy consumption of LPNs and improves the transmission efficiency of the friendship mechanism. Its main contributions are as follows:

- Load prediction of friend nodes: To address the energy consumption of LPNs, the network load of friend nodes is predicted using the Seasonal Autoregressive Integrated Moving Average (SARIMA) model. According to the prediction results, the Receive Window (RW) is adjusted to react to the real-time load of the node in network operation;
- Lightweight GBN protocol: To further reduce the energy consumption of LPNs, a lightweight GBN protocol is designed to simplify the ACK mechanism and windowing mechanism of the GBN protocol.

The rest of this paper is organized as follows: Section 2 focuses on the simulation model of the BLE Mesh network, Section 3 describes the detailed design of the TP-LW algorithm, Section 4 explains the simulation experimental configurations and their results, and finally, in Section 5, conclusions are given.

## 2. System Model

**Figure 1:**
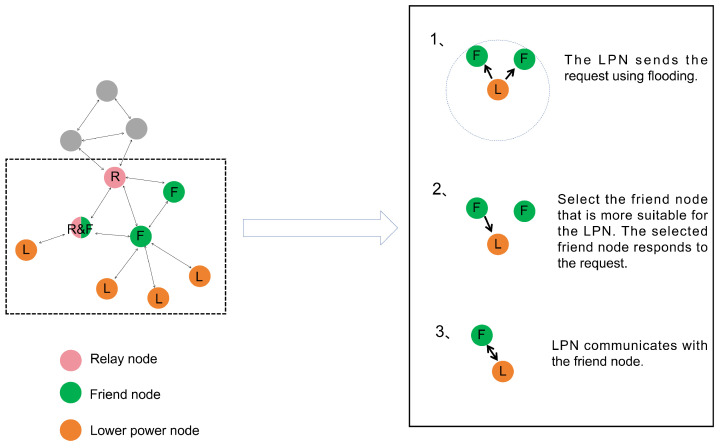
Bluetooth mesh node types.

This section focuses on the system model of the BLE Mesh. The BLE Mesh standards are taken from the Bluetooth website (www.bluetooth.com, accessed on 9 July 2024). As shown in Figure 1, there are different types of nodes present in a BLE Mesh network. They have special functions in the network [4]. The four most important features are the Relay feature, the Friend feature, the low-power feature, and the Proxy feature. Nodes can have one or more features, but low-power features cannot coexist with other features. This is because nodes with low-power features need to be dormant for a long time and cannot fulfill other functions. When a node has both relay and friend characteristics, it can forward packets in the network. It can also establish a friend relationship with LPNs and send packages to LPNs in the network. In the BLE Mesh network, relay nodes will use a constrained flooding technique to broadcast information. The process of establishing a friendship relationship is also shown in Figure 1. The friend node will cache the received packets for the LPN that has established a friendship relationship. When the LPN is in a wake-up state, it will request the cached packets from the friend node. During this period, the LPN communicates with the friend node using the stop-and-wait protocol.

### 2.1. Network Load Modeling

In the BLE Mesh system model, the first step is to model the network load of the friend node. We calculate the network load of a node per unit time by the length *l* of the send queue. The packets cached in the send queue are divided into two parts. One part comes from the packets to be forwarded in the network $l_{r}$. The other part comes from the packets $l_{lpn}$ requested by the LPN after it wakes up. Thus, the number of packets $l_{t}$ queued per unit of time can be expressed as

(1) $$l_{t}=l_{r}+l_{lpn}$$

A friend node can establish friendship relationships with multiple LPNs. These LPNs wake up periodically. When an LPN wakes up, it requests the packets cached for it during hibernation from the friend node that established the friendship relationship. During the wake-up cycles of the LPNs, the length of the sending queue of the friend nodes will increase significantly. Thus, the network load on friend nodes is significantly temporal and seasonal. Friend nodes, as IoT devices, have limited computing power, and it is challenging to execute complex time series prediction algorithms with them. We consider the characteristics of the network load of friend nodes, the computing power of devices, and the advantages and disadvantages of different time series prediction algorithms. Therefore, we adopt the SARIMA model [20] of classical time series prediction algorithms to predict the network load of friend nodes.

The SARIMA model evolved from the ARIMA (Autoregressive Integrated Moving Average Model) model, which consists of the Autoregressive Model (AR), Single-Integer Order *i*, and Moving Average Model (MA). The ARIMA (*p*, *d*, *q*) model is expressed as

(2) $$y_{t}=\alpha +\sum_{i=1}^{p}\gamma_{i}\epsilon_{i-1}+\epsilon_{t}+\sum_{i=1}^{q}\theta_{i}\epsilon_{t-i}$$

where $y_{t}$ is the current term, α is the constant term, *p* is the autoregressive term, $\gamma_{i}$ is the autocorrelation coefficient, ϵ is the residual series, *q* is the moving average term, $\theta_{i}$ is the coefficient of the moving average term, and *d* is the number of differencing performed when the time series are smoothed.

The ARIMA model only considers autocorrelation and trends in the time series and cannot capture the effect of seasonality such as LPN wake-up cycles. Therefore, a seasonal ARIMA model is required. The SARIMA model adds three hyperparameters, *P*, *D*, and *Q*, to the ARIMA model, as well as an additional seasonal cycle parameter *S* [21]. It is able to use pattern recognition, estimation, and forecasting procedures of the Box–Jenkins method. Facilitating real-time model adjustments as more historical data become available, the general expression for SARIMA (*p*, *d*, *q*)(*P*, *D*, *Q*) *S* is

(3) $$\Phi_{p}\left(L\right)\Phi_{p}\left(L^{S}\right)\Delta^{d}\Delta_{S}^{D}y_{t}=\Theta_{q}\left(L\right)\Theta_{Q}\left(L^{S}\right)\epsilon_{t}$$

where *L* is the lag operator, *P* is the maximum lag order of the seasonal autoregression, *Q* is the maximum lag order of the periodic moving average operator, and *D* is the number of seasonal differences.

### 2.2. Friend Node Selection

In the process of friend node selection, each candidate friend node will calculate its own Local Delay based on the parameters and RSSI (Received Signal Strength Indication) in the friend request message. This allows LPNs to distinguish between friend nodes of different performance when selecting a friend. It also avoids broadcast conflicts when multiple friend nodes reply to friend request messages at the same time. After the delay has elapsed, it will respond to the friend request message to the LPN. The formula for the calculation of the Local Delay is as follows:

(4) LocalDelay=ReceiveWindow×ReceiveWindowFactor−RSSI×RSSIFactor

According to the BLE Mesh standard protocol, the ReceiveWindow parameter ranges from 1 to 255 ms, reflecting the network load of the friend node. RSSI is measured by the friend node, reflecting the communication status between the friend node and the LPN. The ReceiveWindow Factor and RSSI Factor are set by the LPN informing the friend node through the friend request message. The standard protocol specifies that these values can be 1, 1.5, 2, and 2.5. By the different settings of the two factors, the friend node that better meets the needs of the LPN can be prioritized and filtered out.

When a friend node receives a request to establish a friendship relationship, it will calculate the length of its sending queue according to the result of the network load predicted by the SARIMA model and adjust the Receive Window of the friend node according to Equation (5):

(5) $$ReceiveWindow=T_{d}\times \frac{1}{n}\times \sum_{i=1}^{n}l_{t+i}$$

where $T_{d}$ is the time for a friend node to complete the transmission of a packet on three broadcast channels during a normal operating state. The friend node obtains the length of its send queue when *n* low-power nodes request it by SARIMA model prediction, calculates the average value, and multiplies it with the time $T_{d}$ to obtain the ReceiveWindow value. The computed Receive Window reflects the network load of the friend node when the LPN wakes up.

### 2.3. Friendship Mechanism Communication Model

**Figure 2:**
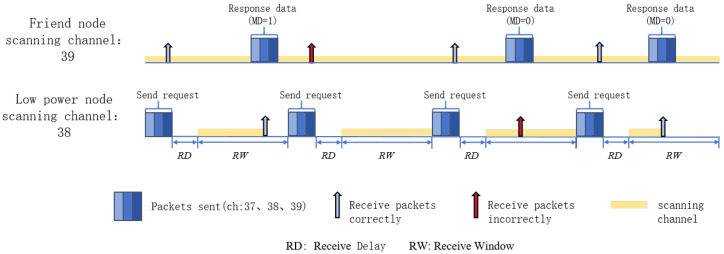
BLE Mesh network friendship mechanism communication example.

The BLE Mesh standard protocol uses a stop-and-wait protocol for data transmission between LPNs and friend nodes, and the process is shown in Figure 2.

After the relationship is established between the LPN and the friend node, the LPN goes to sleep. Then, it wakes up periodically and sends a friend polling message to the friend node to request the packets cached during the sleep period to be sent to itself. In Figure 2, the friend node caches two packets for the LPN at this time and sends the first packet to the LPN after receiving the friend polling message. After receiving the response from the friend node, the LPN can briefly enter the sleep state and wait until the Receive Window expires to send the request again to reduce the scanning time. However, when the friend node cannot correctly receive the request packet, or the LPN cannot correctly receive the message replied to by the friend node, the LPN will scan the Receive Window time. When the MD field in the packet is 0, it means that there is no more packet cached for the LPN at the friend node. The LPN can enter the sleep state and wait for the next wake-up cycle.

There are three states of LPNs in this process, sleep, sending packets, and scanning. In [22], the energy consumption in three states of LPNs in the BLE Mesh network has been tested and calculated to obtain the energy consumption in each state, as shown in Table 1.

From Table 1, it can be concluded that the energy consumption of an LPN is mainly composed of three parts. The first part is to keep working even while the node is dormant, such as the timer responsible for periodically waking up the LPN and the peripheral devices responsible for monitoring or collecting information. The second part is the energy consumed by polling the friend node to request packet delivery during wake-up. The third part is the energy consumed by the channel scanning of the LPN during the wake-up cycle. Thus, the energy consumption of the LPN can be expressed as

(6) $$E=T_{sleep}\times I_{sleep}+R_{eq}\times I_{send}+T_{receive}\times I_{receive}$$

where *E* denotes the total energy consumption of the LPN. $T_{sleep}$ denotes the time when the LPN is in the sleep state. $I_{sleep}$ denotes the energy consumed per millisecond when the LPN is in the sleep state. $R_{eq}$ denotes the number of polling packets sent by the LPN to the friend node. $I_{send}$ denotes the energy consumed by the LPN to send a polling packet. $T_{receive}$ denotes the time when the LPN is scanning, and $I_{receive}$ denotes the energy consumed per millisecond when the LPN is scanning.

## 3. Algorithm Design

This section describes the details of the TP-LW algorithm. The TP-LW algorithm first predicts the load of friend nodes by the SARIMA model. Then, based on the prediction result, it optimizes the selection of friend nodes so that the LPN establishes a friendship relationship with the optimized friend node. Finally, the nodes communicate with each other based on the lightweight GBN protocol.

### 3.1. Load Prediction for Friend Nodes

**Figure 3:**
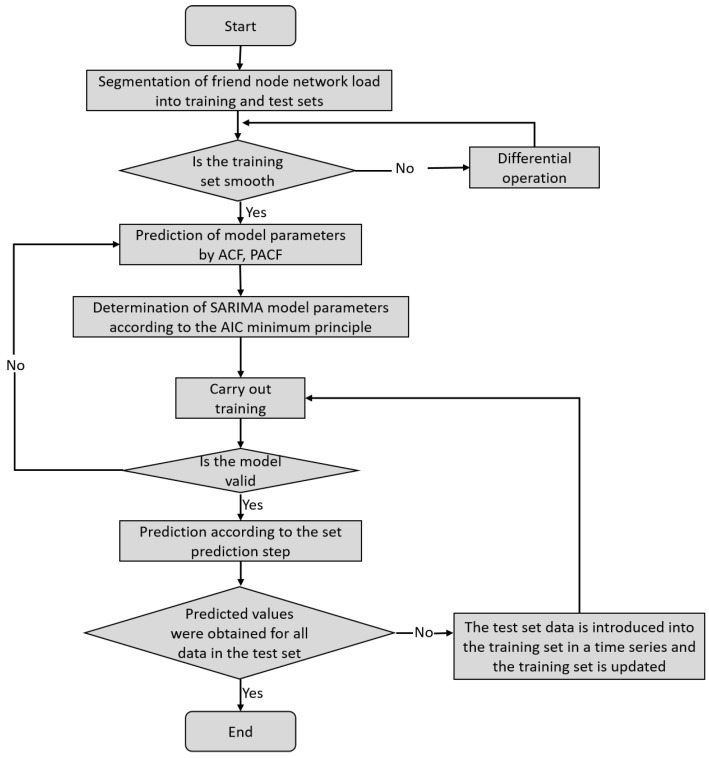
Flowchart of SARIMA time series forecasting model.

The TP-LW algorithm firstly predicts the network load of friend nodes based on the SARIMA (*p*, *d*, *q*) (*P*, *D*, *Q*) *S* model, which mainly includes four steps: time series smoothing processing of network load of friend nodes, model parameter identification, model checking, and model prediction, and the specific framework is shown in Figure 3. The ACF (Autocorrelation Function) is used to determine the degree of autocorrelation of the time series. The PACF (Partial Autocorrelation Function) is used to determine the degree of direct correlation between the two time series after eliminating the influence of other lag terms. The AIC (Akaike Information Criterion) is used to assess the degree of fit and generalization of the SARIMA model. For the time series $x_{t}$ with lag order k of the series $x_{k-t}$, the ACF and PACF are calculated as follows:

(7) $$c_{k}=\frac{1}{n}\sum_{t=1}^{n-k}\left(x_{t}-\bar{x}\right)\left(x_{t+k}-\bar{x}\right)$$

(8) $$ACF_{k}=\frac{c_{k}}{c_{0}}=Cor\left(x_{t},x_{t+k}\right)$$

(9) $$PACF_{k}=\left\{\begin{array}{cc} Cor\left(x_{1},x_{0}\right)=r_{1} & if\;k=1;\\ Cor(x_{k}-x_{k}^{k-1},x_{0}-x_{0}^{k-1}) & if\;k\geq 2; \end{array}\right.$$

Friend nodes are first divided into training and test sets by the historical series of network loads for prediction using the SARIMA model. The Augmented Dickey–Fuller (ADF) test is used to determine whether the initial time series is smooth. If it fails the ADF test, the series is not smooth enough. This requires the conversion of the nonstationary time series into a stationary time series by a difference operation. Then, the parameter set for predicting the load sequence of the friend node network is determined by combining the ACF and PACF methods. The optimal parameters of the model are determined by the AIC criterion method. Before using the model to make predictions, the validity of the model needs to be tested. This is achieved by looking at the residuals of the SARIMA model and observing the correlation of the residuals. If the residuals are close to normal distribution and not correlated, the model is valid. Finally, the network load sequence is predicted by the obtained model.

We use two metrics to measure the forecasting performance of the SARIMA model, which are Root Mean Squared Error (RMSE) and Mean Absolute Error (MAE). RMSE is sensitive to very large or very small errors in the prediction results and can reflect the accuracy of the prediction very well. MAE can better reflect the actual situation of the prediction error because the error is taken as an absolute value, and there is no positive or negative offset. The calculation formulas are shown below:

(10) $$RMSE\left(y,\hat{y}\right)=\sqrt{\frac{1}{\left|N\right|}\sum_{i\in N}{\left(y_{i}-{\hat{y}}_{i}\right)}^{2}}$$

(11) $$MAE\left(y,\hat{y}\right)=\frac{1}{\left|N\right|}\sum_{i\in N}\left|y_{i}-{\hat{y}}_{i}\right|$$

where $y_{i}$ represents the actual network load, ${\hat{y}}_{i}$ represents the predicted value, and *N* represents the number of predicted values. For RMSE and MAE, the lower the value of the evaluation metric, the more accurate the prediction.

### 3.2. Lightweight GBN Protocol

**Figure 4:**
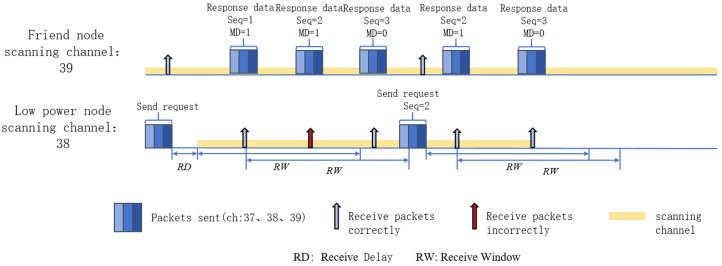
Lightweight GBN protocol communication schematic.

The second key point of the TP-LW algorithm is the lightweight GBN protocol. In our designed lightweight GBN protocol, the LPN only needs to send a friend polling message once at the initial startup of each wake-up cycle, and it informs the friend node about the sequence number of the latest packet it received in case of packet loss. After receiving the friend polling message, the friend node will send the packets to the LPN in an orderly manner according to a certain broadcast interval. When the LPN successfully receives the packets sent by the friend node, it will immediately reset its Receive Window and continue to receive the packets until it receives all the packets cached by the friend node. These designs can reduce the number of packets sent by LPNs, shorten the scanning time, and reduce the energy consumption caused when switching states. Figure 4 shows the transmission mechanism of the lightweight GBN protocol.

The friend node sends the packet by a sequence number (Seq). When packet loss occurs, the Seq received by the LPN will no longer be in order. At this point, the LPN sends a friend polling message again to inform the latest packet the friend node received correctly. After receiving the friend polling message, the friend node will resend the packet that the LPN has forfeited. Selective Retransmission (SR) protocols can also be used for such communication, but the receiver of the SR needs to cache all received packets and process the Seq of the packets. This is not conducive to reducing the energy consumption of LPNs. In contrast, LPNs, as receivers in the GBN protocol, only need to maintain Seq information.

### 3.3. Overall Flow Chart of TP-LW Algorithm

**Figure 5:**
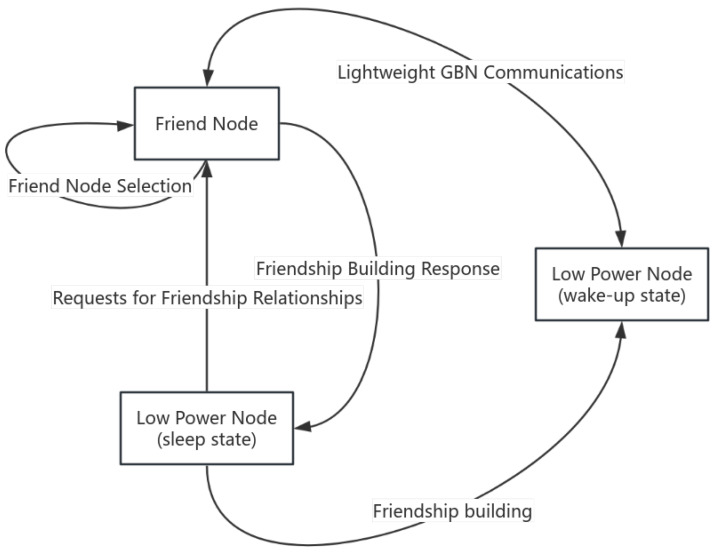
Constrained flooding algorithm based on time series prediction and lightweight GBN flow chart.

The flow of the TP-LW algorithm is shown in Figure 5. The LPN in sleep state sends a friend request message to the nodes in range. The node that receives the friend request will use the time series prediction model to predict its load condition and update the Local Delay. After selecting the friend node, a response message is sent to the LPN. Then, after receiving the response message the LPN enters the wake-up state and communicates with the friend node using the lightweight GBN protocol.

**Figure 6:**
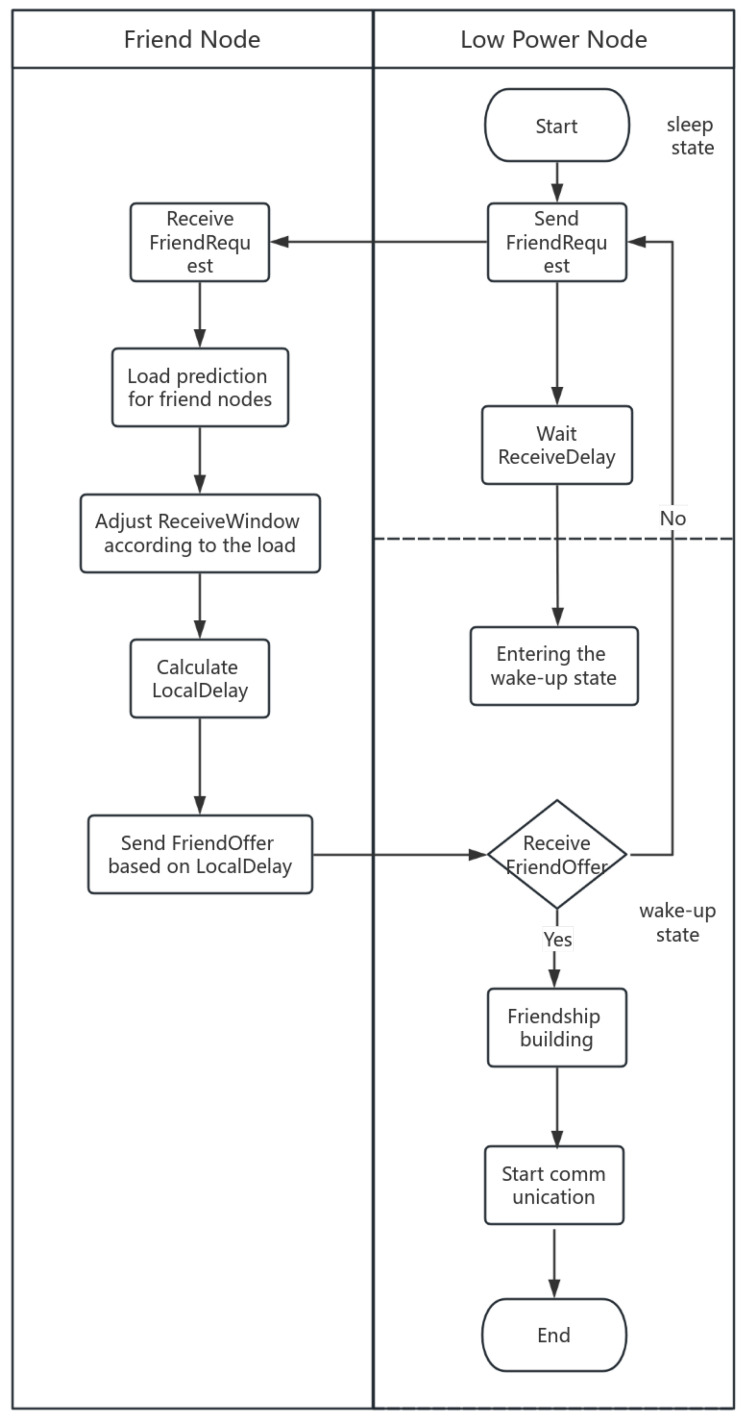
Load prediction and friend node selection.

Figure 6 shows the flowchart about load prediction and friend node selection. First, the LPN sends a FriendRequest to the nodes in the range. Then, the nodes receive the request, predict their load situation, and update ReceiveWindow and Local Delay. The better the load condition, the lower the Local Delay of the node, and the earlier it will reply to the LPN with the FriendOffer of the friendship relationship establishment. If the LPN receives the FriendOffer, it establishes a friendship relationship with the friend node and communicates with the friend node in the wake-up state. If the LPN does not receive the FriendOffer, it waits for ReceiveDelay and then sends the FriendRequest message again.

**Figure 7:**
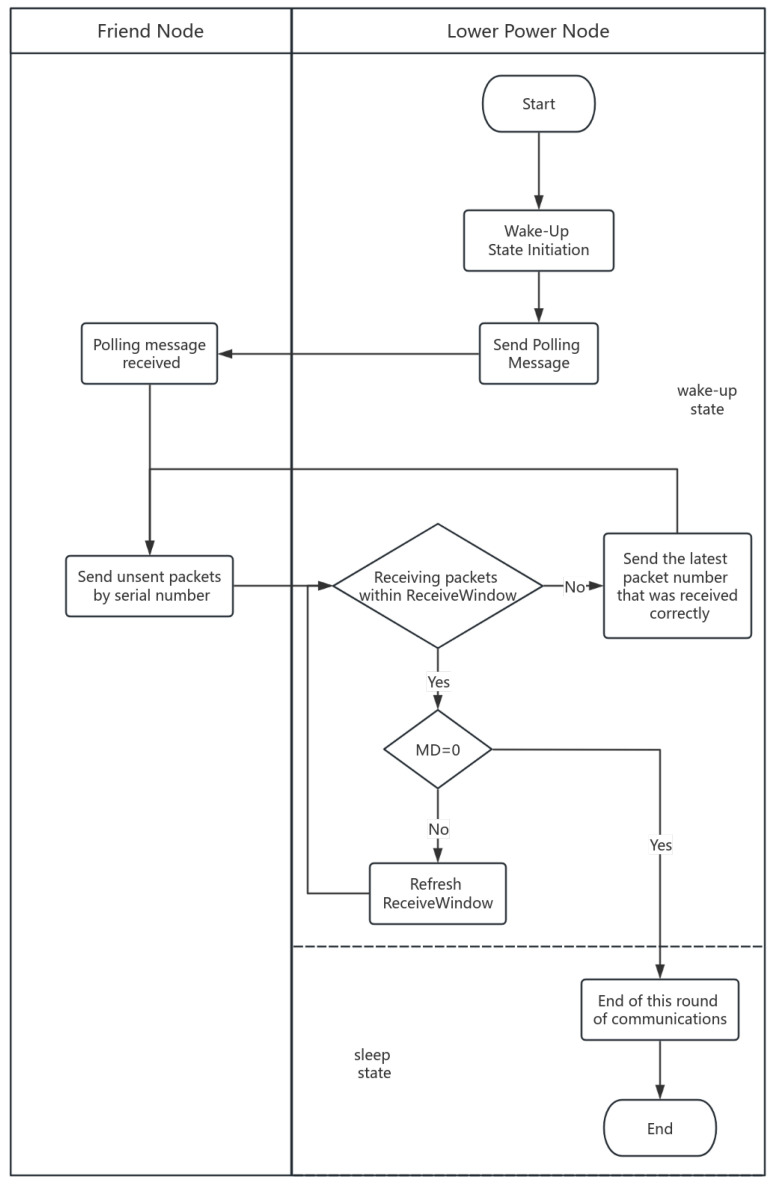
Lightweight GBN communications.

Figure 7 shows the flowchart of lightweight GBN communication. When the LPN is in the wake-up state, it sends a polling message to the friend node to request the packets cached during the sleep state. After receiving the polling message, the friend node sends packets to the LPN in order. If the LPN receives the packet within the ReceiveWindow, it looks at the MD field, and MD = 0 means that this is the last packet that the friend node cached for the LPN. If MD is not 0, the LPN refreshes the ReceiveWindow and waits for the packet to be transmitted. If MD = 0, the LPN will end this round of communication and enter the sleep state. In addition, if the LPN does not receive the packet within the ReceiveWindow, it will send the latest Seq to the friend node for the friend node to resend it.

## 4. Simulation Results

### 4.1. Simulation Experimental Design

This section describes the set-up of the simulation experiment scenario and the setting of network parameters. Due to the complexity of developing a real testbed, the simulation results are the main benchmark for wireless networks [23]. In this paper, network simulation is also used to evaluate the effectiveness of the algorithmic scheme. The simulation is carried out through dedicated software developed in Python [24]. The simulator implements the basic functions of different layers in the BLE Mesh network. The scene consists of an LPN that has just joined the BLE Mesh network and several nodes that are already in the network. The number of friend nodes that have established friendship relationships is randomly and uniformly generated in the interval [0,4], aiming to simulate the random distribution of friend nodes in the real scenario. Meanwhile, this paper sets the number of packets forwarded by friend nodes in the network according to the Poisson distribution model. As for the LPNs that have established friendship relationships, the number of packets cached for them by the friend nodes in each wake-up cycle is also determined based on the Poisson distribution. The model can accurately reflect the random transmission of packets in a real network environment.

The detailed parameter configuration of the experiment is shown and explained in detail in Table 2.

In the dataset collection session, the friend node records the number of packets in the send queue every 100 ms, and the data recording continues for 10 s. The collected data are divided into the ratios of 80% and 20%, where 80% of the data are used as a training set, and the remaining 20% of the data are used as a test set to verify the accuracy of the model predictions.

### 4.2. Simulation Analysis

**Figure 8:**
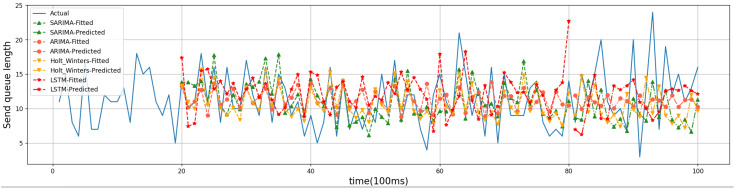
Friend node #1.

**Figure 9:**
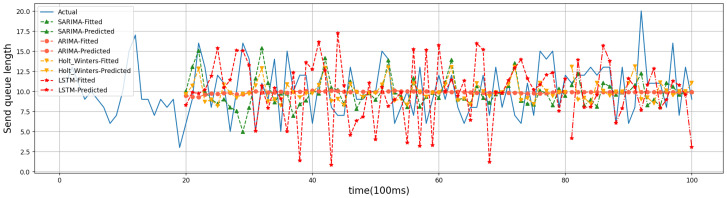
Friend node #2.

**Figure 10:**
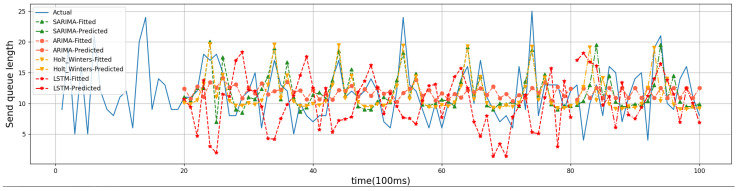
Friend node #6.

**Figure 11:**
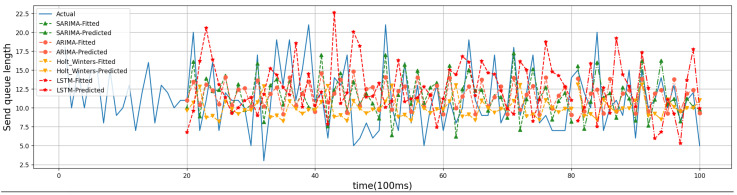
Friend node #7.

For the evaluation of the time-series prediction models, we compared the ARIMA, Holt–Winters [26], and Long Short-Term Memory (LSTM) models [27]. Holt–Winters and LSTM are both classical time series prediction models that are used as comparison algorithms to show the prediction effect of SARIMA. The training dataset for Holt–Winters and LSTM is the same as that for SARIMA in that 80% of the historical data are used as the training set and 20% as the test set. Below are four randomly generated friend nodes that have established friendship relationships with different numbers of LPNs. The results of time series forecasting can be better demonstrated. Friend nodes #1, #2, #6, and #7 have established friendship relationships with 3, 1, 4, and 2 LPNs, respectively. The four models were trained on the first 8 s of data, predicted the trend of the last 2 s, and compared with the actual values. Figure 8, Figure 9, Figure 10 and Figure 11 show the predicted graphs of sending queues for friend nodes with different numbers of friendship relationships. We can draw the following conclusions:

- Significant advantages are demonstrated by the SARIMA model in predicting the network load of friend nodes.

When an LPN that has established a friendship relationship with a friend node wakes up, the network load of the friend node increases significantly. The SARIMA model can predict the period when the network load of the friend node increases more accurately. This provides the newly joined LPNs with accurate information when selecting a friend node.

**Figure 12:**
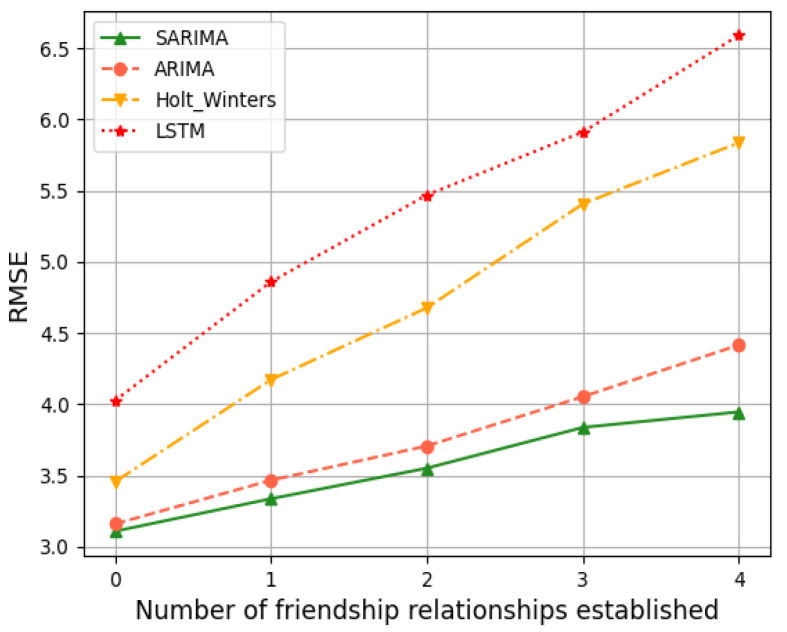
RMSE.

**Figure 13:**
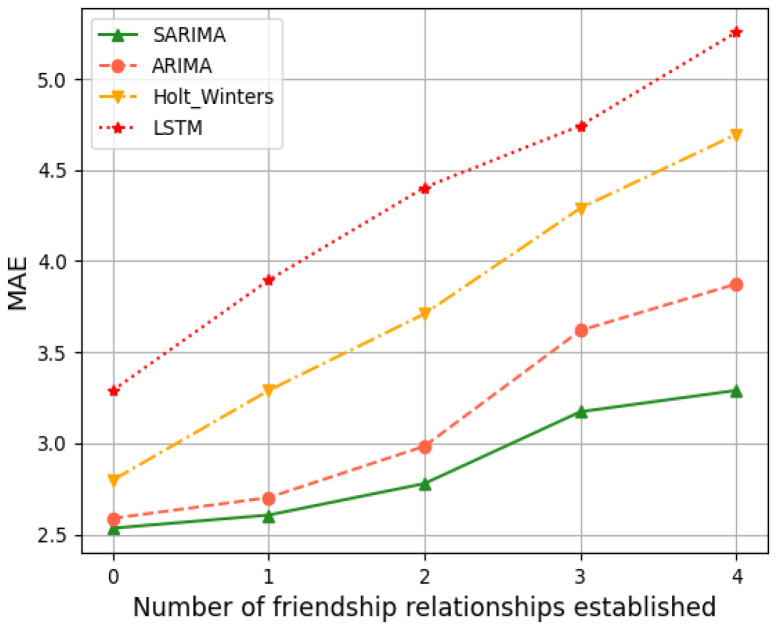
MAE.

To evaluate the general applicability, as well as the accuracy, of the SARIMA model in predicting the network load of friend nodes, we simulated the model 1000 times for friend nodes with different numbers of friendship relationships. A comparative validation was carried out using two evaluation criteria, RMSE and MAE. From Figure 12 and Figure 13 we can understand the following:

- The prediction results of the SARIMA model are significantly better than those of the ARIMA model, the Holt–Winters model, and the LSTM algorithm, with higher accuracy.

When the friend node has not yet established any friendship relationship, there is no significant increase in the period in the time series graph of its sending queue. And there is no seasonality in the time series. So, the RMSE and MAE values of the SARIMA model, ARIMA model, and the Holt–Winters model are similar. As the number of friendships increases, the seasonality in the time series of the sending queue becomes apparent, and the SARIMA model’s predictions become more accurate. The LSTM algorithm has a high degree of learning freedom, but it requires a certain amount of computational power and data volume from the device. Thus, it affects the accuracy of network load prediction on IoT devices with limited computational power and storage resources.

**Figure 14:**
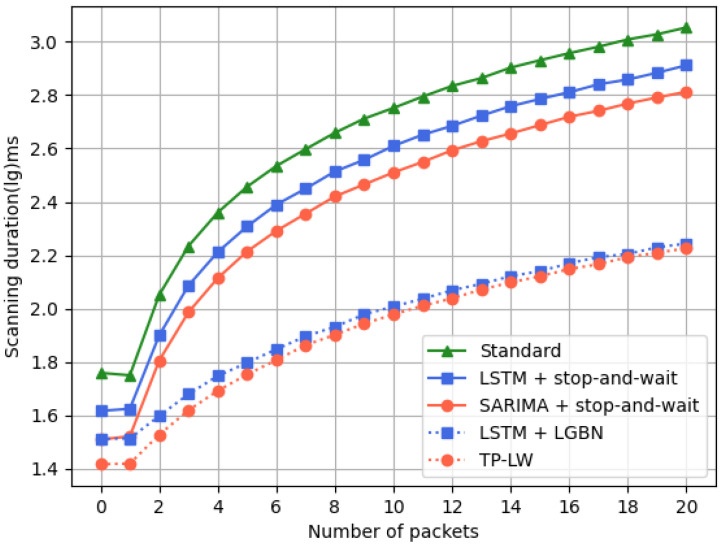
Scanning time.

For the evaluation of the energy consumption of LPNs, we conducted 1000 experiments for networks with different numbers of nodes. The experiments counted the scanning duration of LPNs when obtaining different numbers of packets sent in each wake-up cycle. As can be seen in Figure 14:

- As the number of packets increases, the overall scanning time shows an increasing trend;
- Compared with the standard protocol, both SARIMA and LSTM predictions result in a small reduction in scan time. The LSTM+LGBN and TP-LW algorithms perform better, but the TP-LW algorithm is more effective.

The lightweight GBN protocol only needs to send a request at the beginning of a wake up and when a packet loss occurs. In contrast, the standard protocol requires a request to be sent once for each packet and again when a packet loss occurs. Also, the number of requests that need to be sent using the lightweight GBN protocol increases less when the network environment worsens. This further demonstrates its effectiveness in reducing energy consumption in various network conditions.

**Figure 15:**
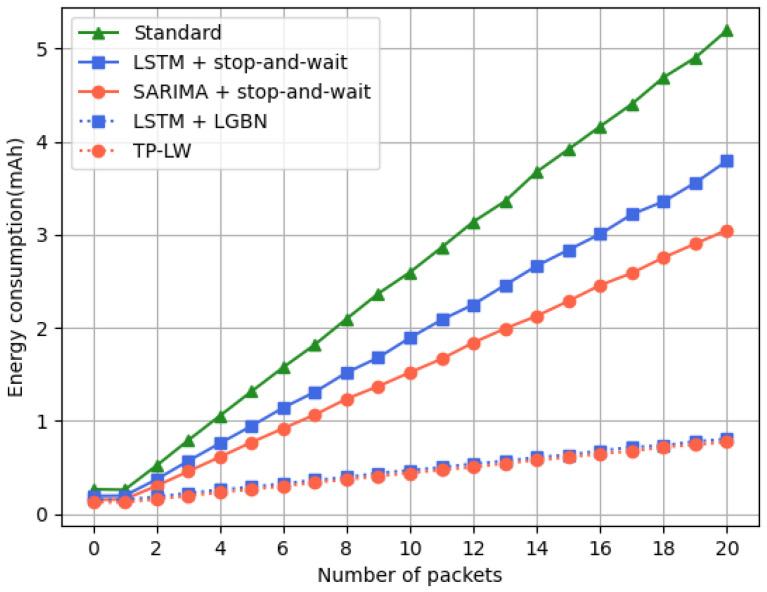
LPN energy consumption.

We calculated the energy consumed by a LPN to receive a specific number of packets in a wake-up cycle. The following conclusions can be drawn from Figure 15:

- The energy consumption of LPN is gradually increasing as the number of packets increases. The prediction with LSTM is better than the standard protocol, while the prediction with SARIMA is even better than LSTM;
- The TP-LW and LSTM+LGBN algorithms produced the best results that were optimal in terms of energy consumption, with the TP-LW algorithm being slightly more effective than LSTM+LGBN.

The network environment deteriorates and the energy consumption of LPNs using standard protocols can increase rapidly. However, the LPNs using the lightweight GBN protocol can maintain a relatively stable energy consumption. The applicability and superiority of the lightweight GBN protocol in different network environments are further verified.

**Figure 16:**
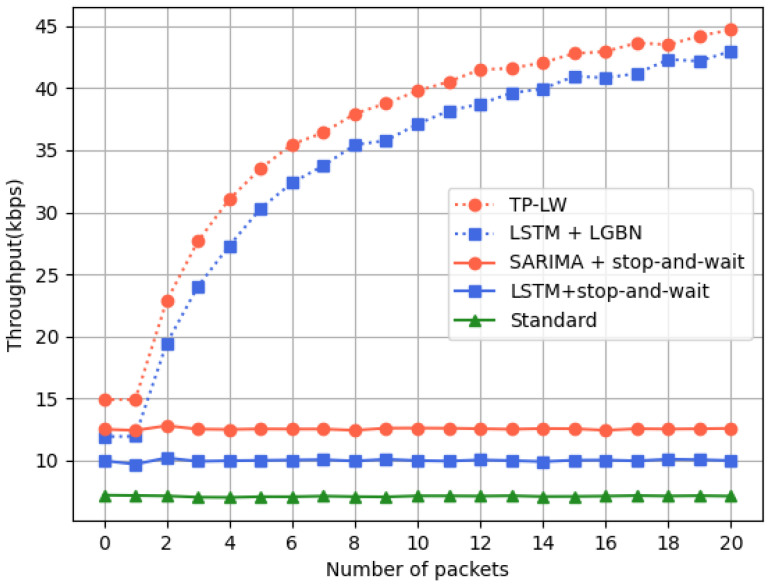
LPNs throughput.

For the evaluation of LPN throughput, we conducted one thousand experiments for different numbers of nodes. The standard protocol, the LSTM+stop-and-wait, SARIMA+stop-and-wait, LSTM+LGBN, and TP-LW algorithms are compared. The results can be seen in Figure 16:

- The throughput using the stop-and-wait protocol remains essentially unchanged. The use of the prediction algorithm outperforms the standard protocol;
- As the amount of data increases, the processing speed of the LPN with the lightweight GBN protocol gradually rises and finally tends to stabilize. The performance using SARIMA is better than LSTM.

In the case of using the lightweight GBN protocol, the friend node sends packets consecutively, shortening the entire transmission process. Therefore the number of new packets entering the queue during this period is relatively less. This means that the LPN has to wait less time for the friend node to process other packets while receiving a packet, which in turn improves the packet processing speed. On the contrary, in the standard protocol, even after receiving a packet, the LPN must wait for the Receive Window to end. The whole transmission process takes a long time. The number of newly added packets in the send queue of the friend node will be greater. So, the LPN sometimes needs to wait a long time to receive new packets, which affects throughput.

**Figure 17:**
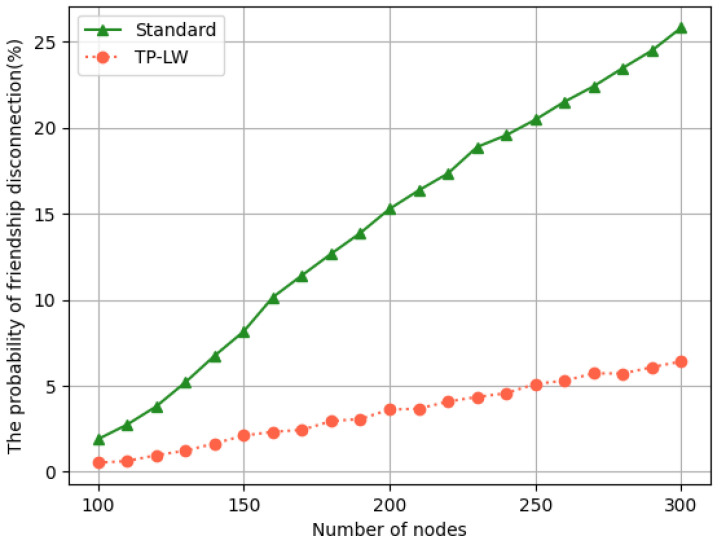
Probability of friendship break.

To assess the stability of the friendship relationship, we compared communication using TP-LW with standard protocols. Since the stability of the friendship relationship comes from the communication process between the LPN and the friend node, the ablation experiments using the same communication protocol have similar results. Only representative TP-LW and standard protocols are shown here. Statistics on the probability of friendship relationship disconnection were performed. The following conclusions can be drawn from Figure 17:

- The use of the lightweight GBN protocol is effective in improving the stability of friendship relationships in networks with varying numbers of nodes.

The lightweight GBN protocol requires only one request to be sent on wake up, whereas the standard protocol uses the stop-and-wait communication method. The LPN needs to request every packet cached during hibernation. Successive loss of requests or loss of replies will lead the LPN to think that the friend node has exited the network and actively end the friendship relationship. Therefore, the stability of friendship relationships is relatively low in the standard protocol.

## 5. Conclusions

In this paper, we propose a constrained flooding algorithm based on time series prediction and lightweight GBN protocol. This algorithm enables energy-limited LPNs to work in a stable state in the network for a long period. On the one hand, the algorithm predicts the network load of friend nodes through the SARIMA model, which provides a reliable basis for LPNs to select a better friend node. On the other hand, the algorithm designs a lightweight GBN protocol to reduce the scanning time of the LPN wake-up state. The energy consumption of LPNs is effectively reduced. Simulation results show that the TP-LW algorithm significantly reduces the energy consumption of the LPNs and improves the throughput of the BLE Mesh network. The TP-LW algorithm enables different types of battery-powered nodes to be integrated into the BLE Mesh network in IoT scenarios. And it also improves the flexibility and diversity of the deployment of the BLE Mesh network.

In our view, the energy consumption of LPNs can be further reduced. In future work, we can optimize the prediction algorithm to extend its use. Prediction can be used not only during the establishment of a friendship relationship but also during each wake-up cycle of LPNs. In addition, packets can be categorized according to the type of service. The prediction results are simultaneously communicated to the LPN using an additional interaction mechanism so that the LPN adjusts the urgency of the wake-up cycles according to the prediction results. This improves the applicability of LPNs to different tasks and enables further reduction in LPN energy consumption. 

## Figures and Tables

**Table 1:** Energy consumption table for each state of the LPN.

State	Symbolic	Energy Consumption (mAh)
Sleep Status	$I_{sleep}$	0.015
Sending Packets Status	$I_{send}$	20.896
Scanning Status	$I_{receive}$	16.057

**Table 2:** Parameters of the friendship mechanism simulation experiment.

Parameter	Value
Number of nodes	$n\in \left[100,300\right],n\in Z^{+}$
Number of friend nodes near LPNs	Randomly selected from the range of 1 to 10
Number of friendship relationships per friend node	Randomly selected from the range of 0 to 4
Physical layer rate	1 Mbps
Node RF range [25]	15 m
Wake-up time	1000 ms
Reception delay	10 ms
Packet length	47 Byte

## Data Availability

Data are contained within the article.
